# Supplementary material for: Wetland fragmentation associated with large populations across Africa
Source: Nat Commun. 2025 May 31;16:5065. doi: 10.1038/s41467-025-59373-2 (PMC12126586; doi:10.1038/s41467-025-59373-2)
Supplement: Supplementary file 1 — Supplementary Information [file 41467_2025_59373_MOESM1_ESM.pdf]

Supplementary Figures for "Wetland fragmentation associated with large populations across Africa"

Sani Idris Garba*[1], Susanna K. Ebmeier[2], Jean-François Bastin[3], Danilo Mollicone[4], Joseph Holden[1]

[1]water@leeds, School of Geography, University of Leeds, Leeds, LS2 9JT, UK;

[2]School of Earth and Environment, University of Leeds, Leeds, LS2 9JT, UK;

[3]TERRA, Teaching and Research Centre, Gembloux Agro Bio-Tech, Université de Liège, Belgium.

[4]Food and Agriculture Organization of the United Nations, Rome, Italy.

***Corresponding author: idrisgarbasani@gmail.com**

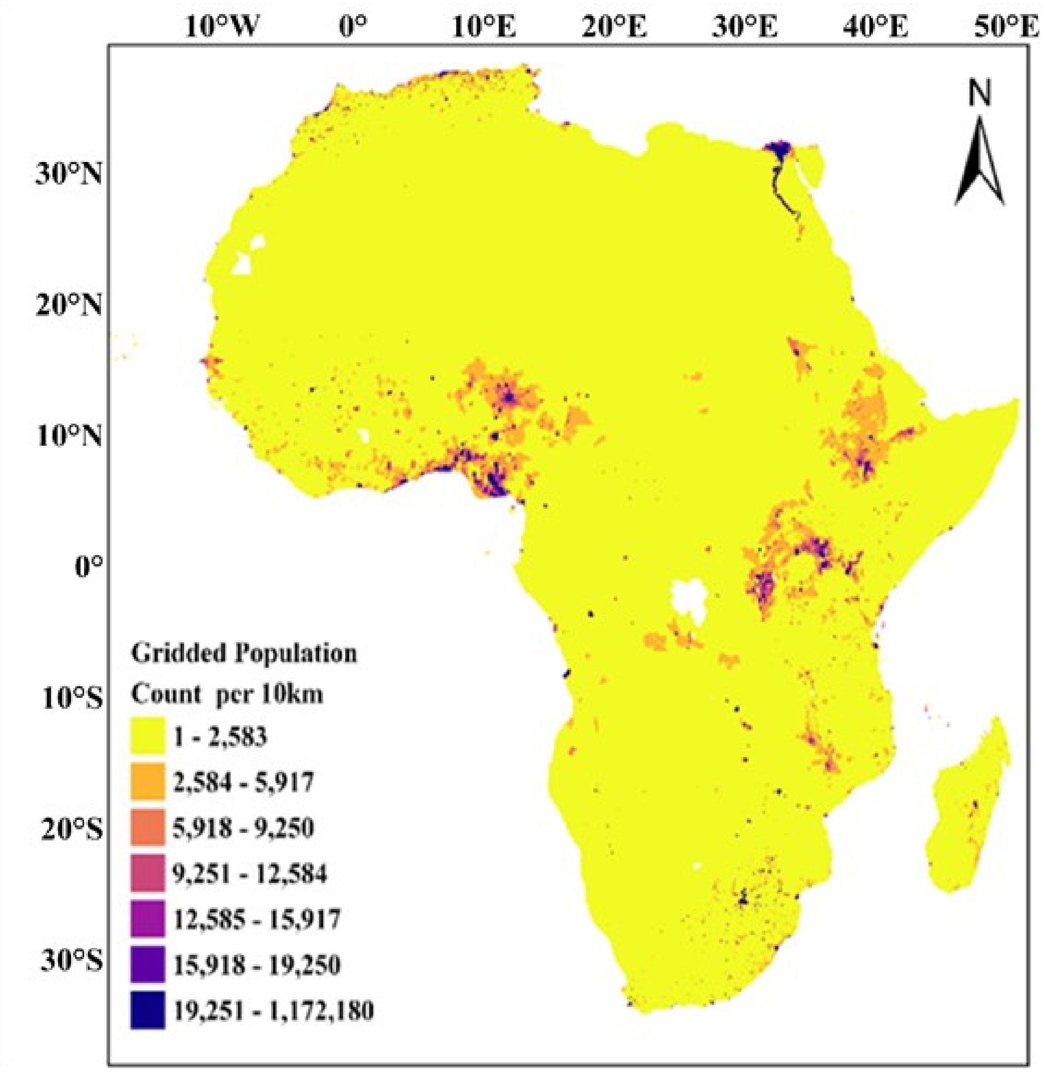

Figure S1. The 10-kilometer square gridded layer of population count of Africa. The yellow regions indicate grids with lowest population count (maximum count of 2,583 persons per 10 km^2), while the ultra blue colored region represents grids with a population count greater than 19,250 persons per 10 km^2. White patches have no data.

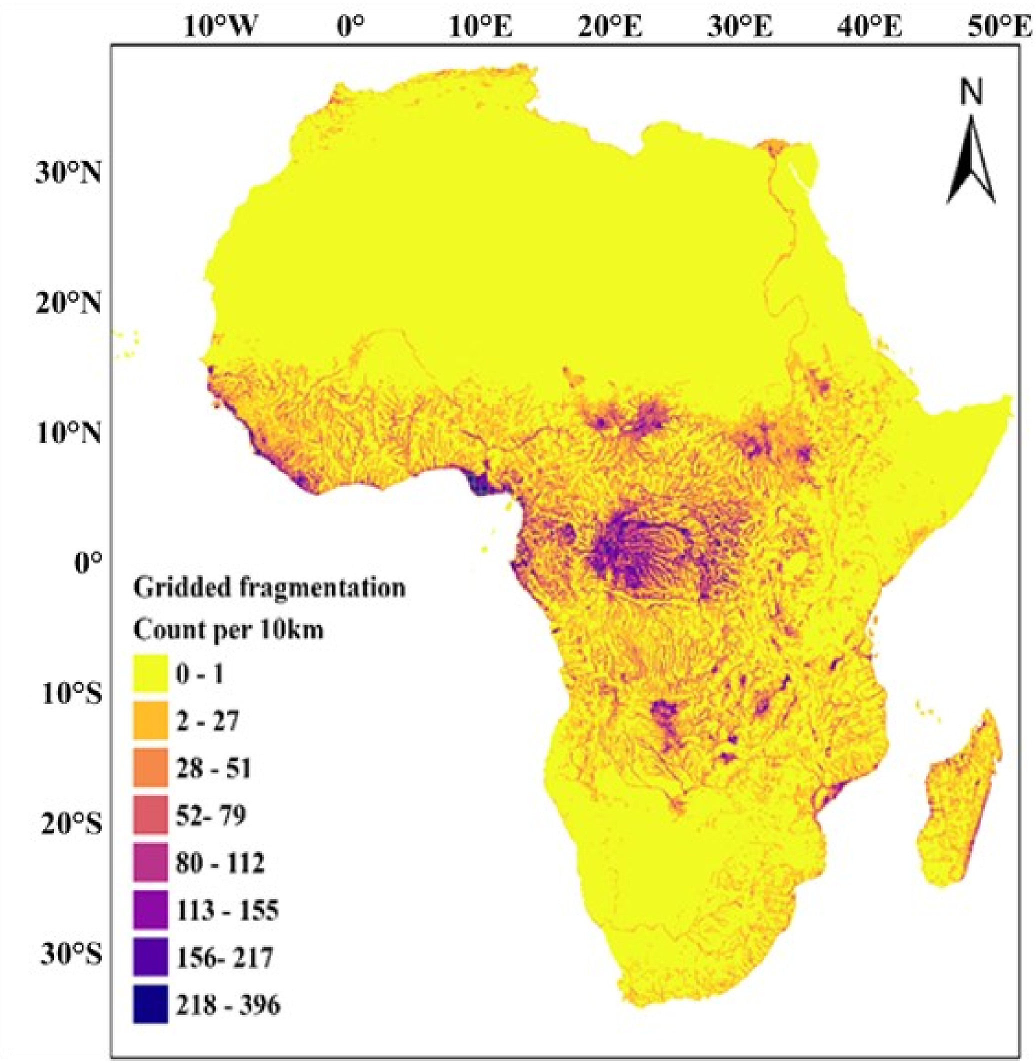

Figure S2. The 10-kilometer square gridded layer of wetland fragmentation in Africa. The yellow regions indicate a non fragmented grid (fragment <=1), while the ultra blue represents a highly fragmented grid.

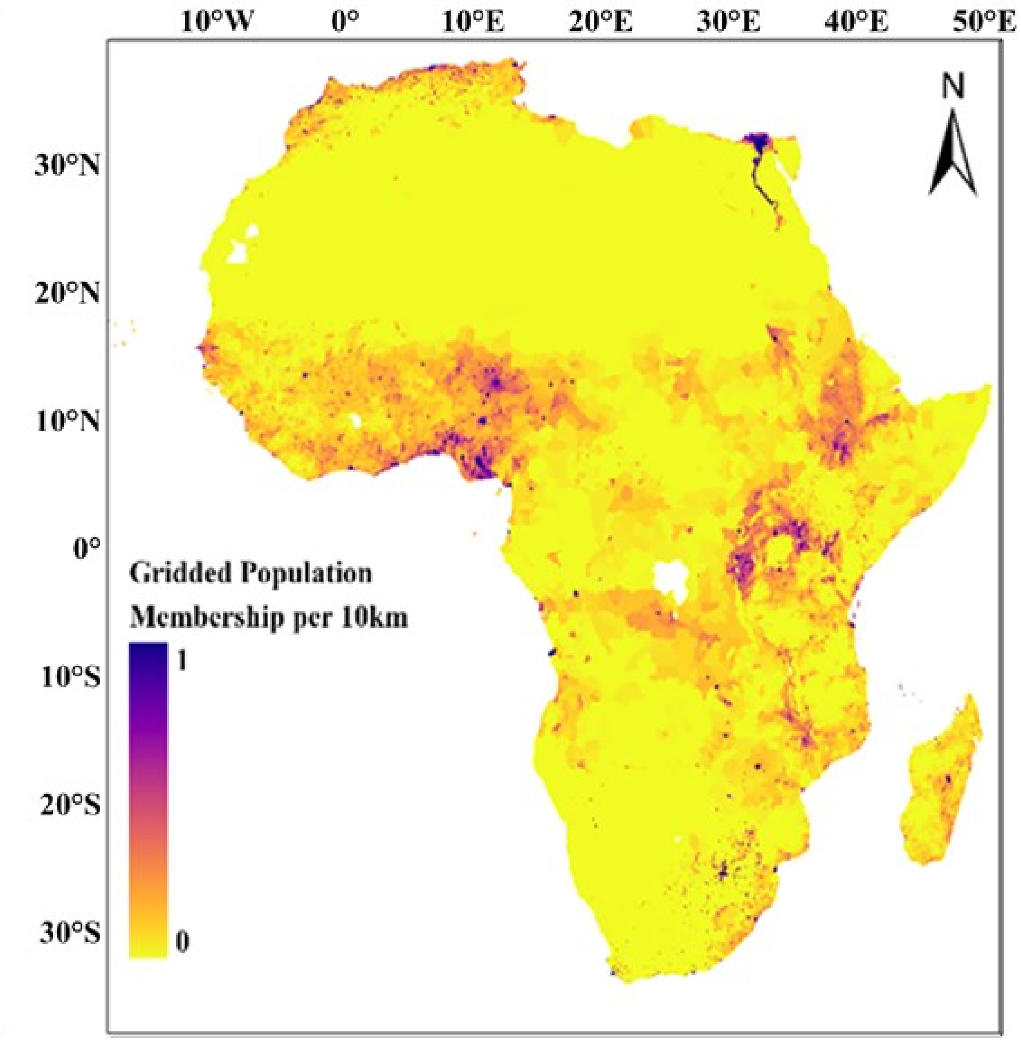

Figure S3. Fuzzy membership showing the distribution from lowest to highest members of the population grid from 0 to 1 in an increasing order. 0 label represents the lowest membership which indicates sparsely populated grids, while the label 1 indicates densely populated grids assigned to the highest membership group.

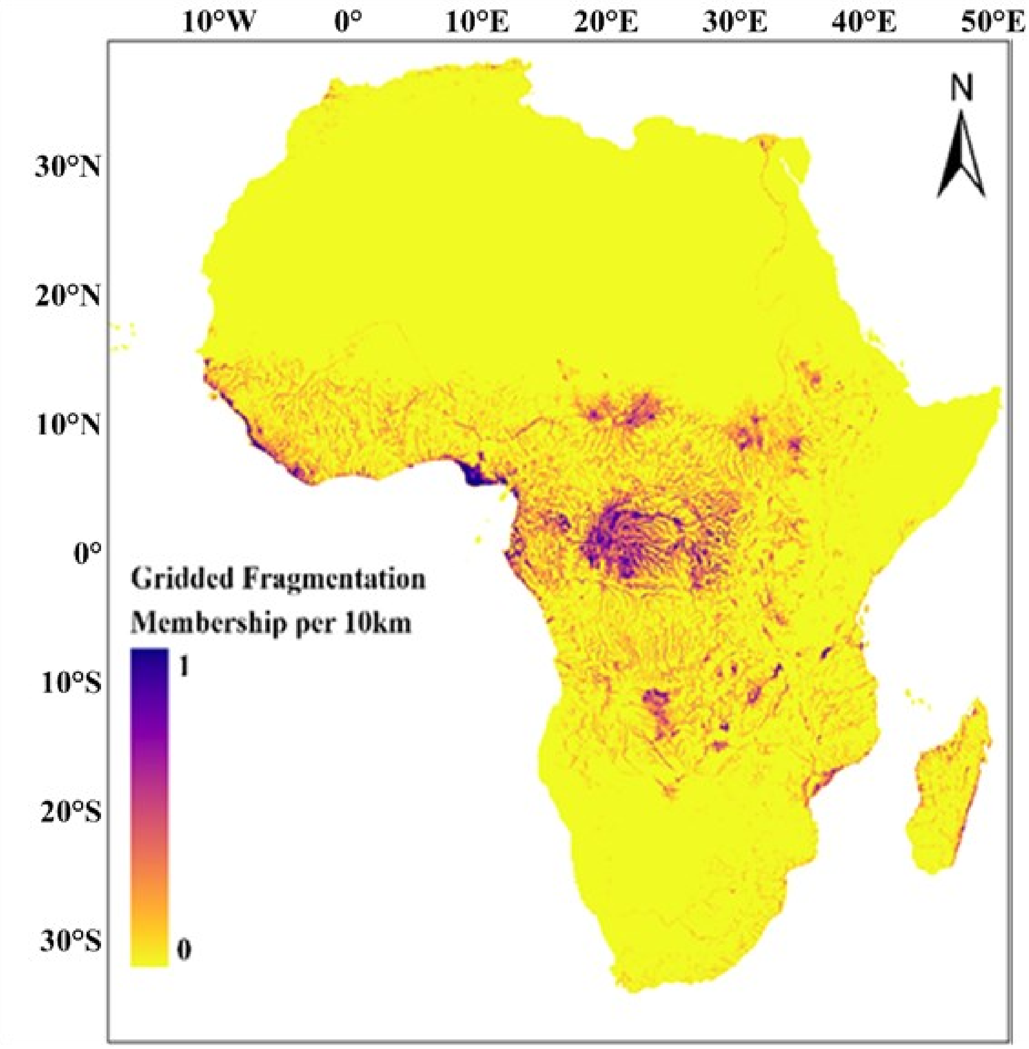

Figure S4. **Fuzzy membership showing the distribution from lowest to highest members of the fragmentation grid from 0 to 1 in an increasing order. 0 label represents the lowest membership which indicates sparsely populated grids, while the label 1 indicates densely fragmented grids assigned to the highest membership group.**

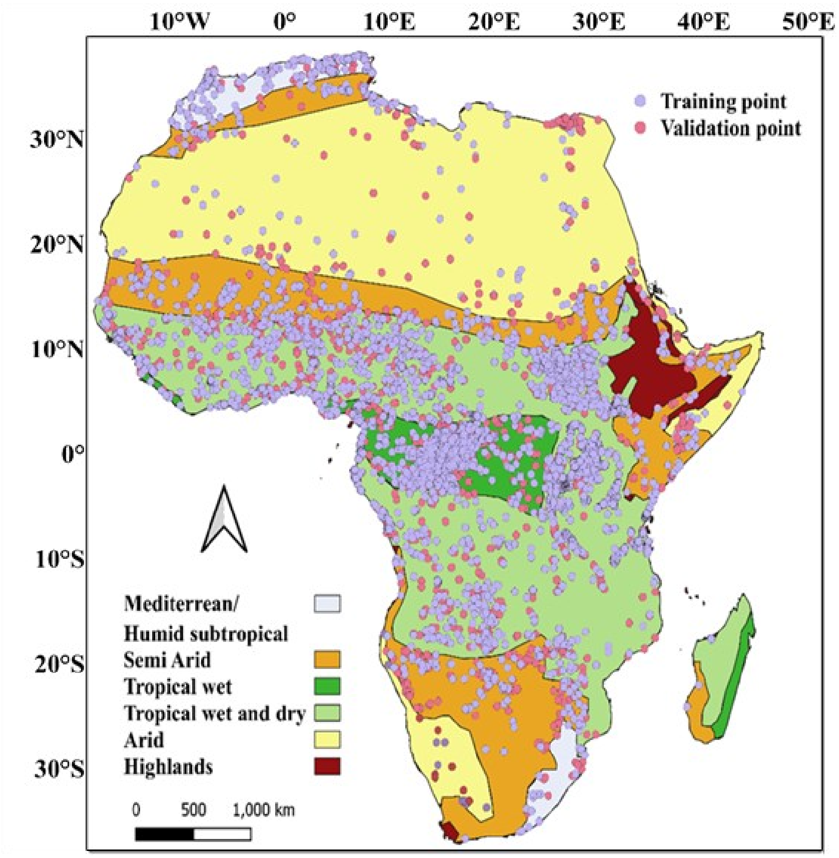

Figure S5. Distribution of training and validation across the different climate zones in Africa. Tropical wet (3,218 points), Tropical wet and dry (2,550 points), Semi-arid (1,144 points), Arid (536) and Mediterranean/humid subtropical (846 points).

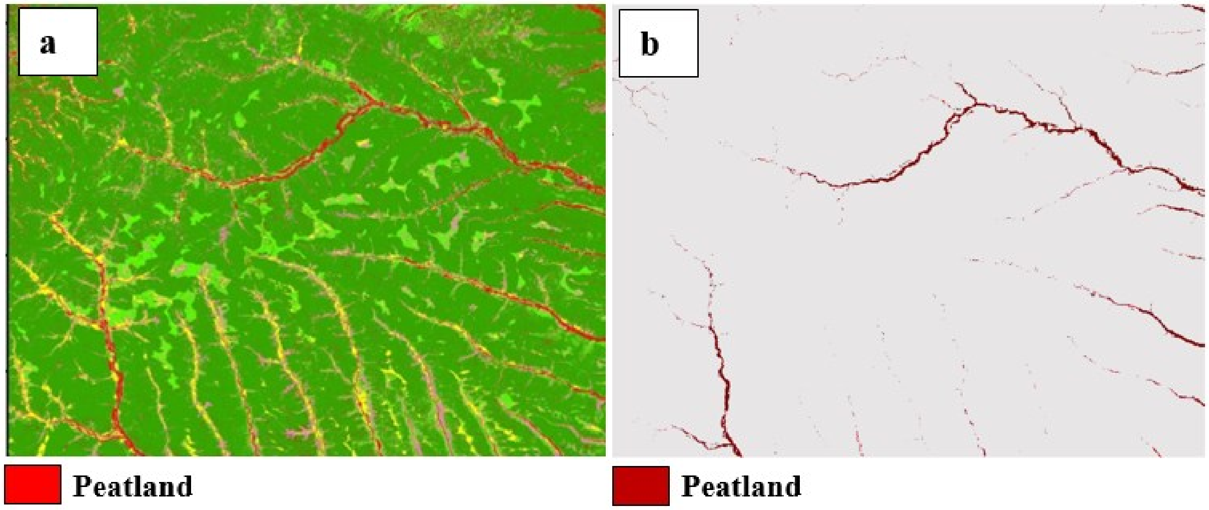

Figure S6. The Map of Angolan highlands peatlands by (a) Lourenco et al. 2022, (b) This study.

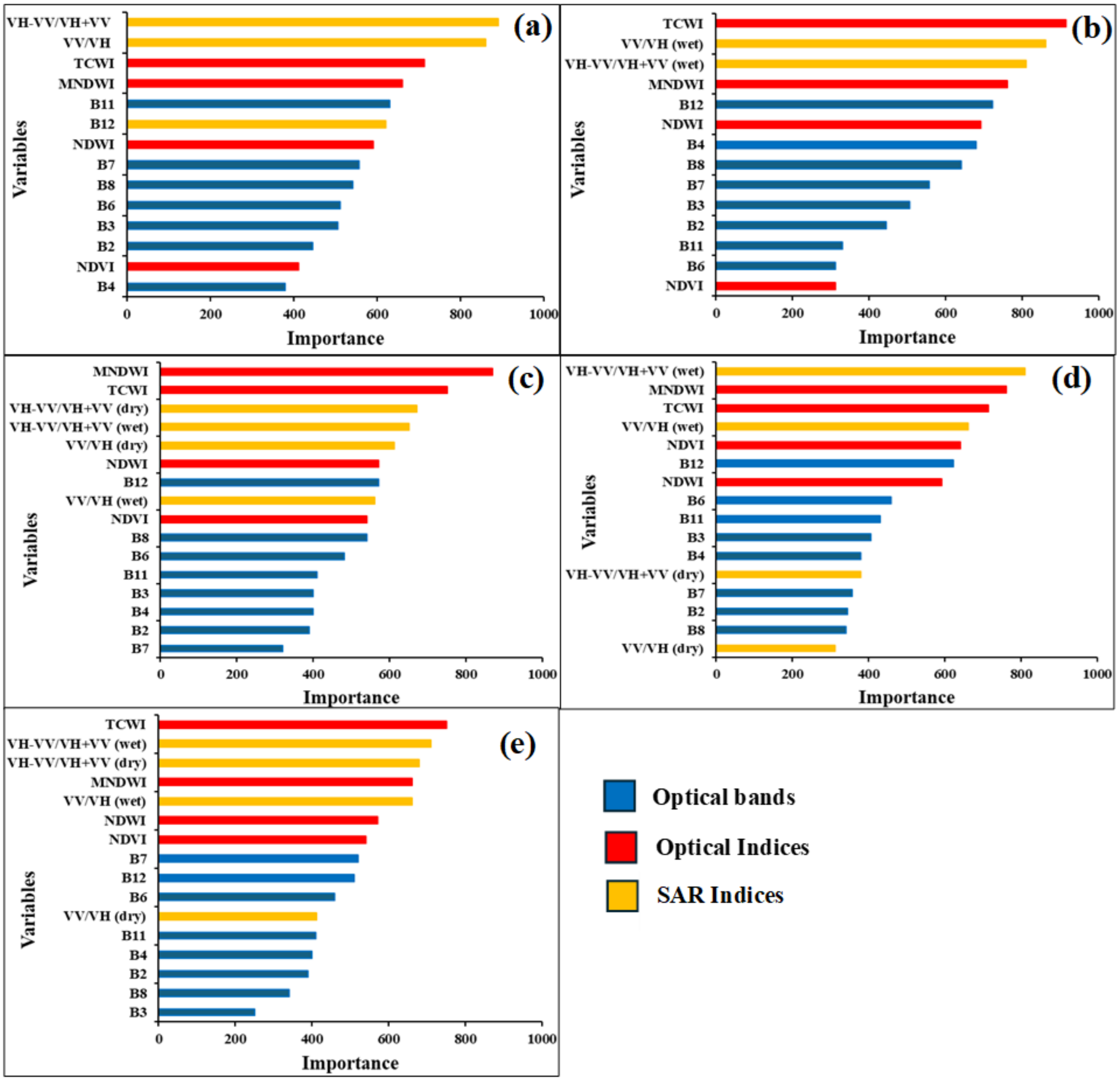

Figure S7. Random Forest variable importance plot extracted from Sentinel-1 and Sentinel-2 features using the training dataset for each climate zone, (a) Tropical wet climate zone, (b)Arid climate zone, (c) Tropical wet and dry climate zone, (d) Semi-Arid climate zone, (e) Mediterranean/Humid subtropical climate zone. The importance of the variable is the sum of decrease impurity each time the variable is selected to be split at the node for the entire trees in the forest and is unitless.

Supplementary Tables for "Wetland fragmentation associated with large populations across Africa"

Table S1 The spatial extent of wetland cover in different African climate zones. Classification of satellite imagery was based on training which was independent for each climate zone.

Climate zone	Total area (km^2)	Wetland area	Percentage wetland cover
**TW**	1,948,865	448,210	23.0
**TWD**	11,302,156	362,980	3.2
**SARD**	6,685,370	93811	1.4
**ARD/DST**	8,962,030	35,853	0.4
**MED/HST**	1,202,970	8,276	0.7

Table S2 The s description of each wetland type used in this study.

**Wetland class**	**Soil systems**	**Water sources**	**Typical settings and features**	**Plant species**
Marsh	Mineral	Direct flow from lakes, streams, precipitation	Edges of lakes and streams, Coastal zone (salt/tidal marshes)	Herbaceous
Swamp	Mineral or Organic	Precipitation, groundwater, freshwater flooding from rivers or lakes	Along large rivers or on the shores of large lakes	Woody, forested
Mangrove	Organic	Precipitation, groundwater and tidal flow.	Coastal zone mostly grows in sheltered low lying coasts estuaries, and lagoons	Trees and shrubs
Peatland	Organic	Groundwater inflow or precipitation	Standing water of lakes or margins of slow flowing rivers,	Herbaceous plants, Shrubs, small trees.
Seasonal wetland	Organic or mineral	Precipitation	Low lying areas and open fields.	Herbaceous

Table S3 Confusion matrix for wetlands of Tropical wet and dry (TWD) climate zone after applying RF.

**Wetland class**	Marsh	Mangrove	Swamp	Peatland	Seasonal wetlands	Deep water	Total	User`s Accuracy
Marsh	510	4	2	0	10	0	526	0.97
Mangrove	4	171	9	6	0	0	190	0.90
Swamp	9	10	141	36	4	0	200	0.71
Peatland	6	4	23	117	0	0	150	0.78
Seasonal wetlands	13	2	2	0	124	6	147	0.84
Deep water	0	0	0	0	12	384	396	0.97
Total	542	191	177	159	153	390	1609	
Producer`s Accuracy	0.94	0.90	0.80	0.74	0.81	0.98		
Overall accuracy (%)								89

Table S4 Confusion matrix for wetlands of Tropical wet (TW) climate zone after applying RF

**Wetland class**	Marsh	Mangrove	Swamp	Peatland	Seasonal wetlands	Deep water	Total	User Accuracy
Marsh	249	0	1	0	5	0	255	0.97
Mangrove	8	120	10	1	6	0	145	0.83
Swamp	10	9	107	29	6	0	161	0.66
Peatland	18	7	65	347	2	0	439	0.79
Seasonal wetlands	26	11	14	14	118	17	200	0.59
Deep water	0	0	0	0	2	73	75	0.95
Total	311	147	197	391	139	90	1275	
Producer Accuracy	0.80	0.82	0.54	0.89	0.85	0.81		
Overall accuracy								84

Table S5 Confusion matrix for wetlands of Semi-Arid climate zone after applying RF

**Wetland class**	Marsh	Mangrove	Swamp	Peatland	Seasonal wetlands	Deep water	Total	User`s Accuracy
Marsh	64	3	7	2	4	0	80	0.80
Mangrove	0	25	4	2	0	0	31	0.81
Swamp	2	3	32	11	2	0	48	0.64
Peatland	2	3	9	34	2	0	55	0.70
Seasonal wetlands	15	2	3	4	99	5	128	0.90
Deep water	0	0	0	0	8	75	82	0.95
Total	83	36	55	53	115	80	424	
Producer`s Accuracy	0.77	0.69	0.58	0.64	0.86	0.94		
Overall Accuracy (%)								79

Table S6 Confusion matrix for wetlands of Arid/Desert climate zone after applying RF

**Wetland class**	Marsh	Mangrove	Swamp	Peatland	Seasonal wetlands	Deep water	Total	User`s Accuracy
Marsh	131	2	4	5	3	0	145	0.90
Mangrove	0	64	5	3	0	0	73	0.88
Swamp	5	9	101	32	6	0	153	0.66
Peatland	2	2	8	35	0	0	47	0.74
Seasonal wetlands	7	2	5	3	40	7	64	0.63
Deep water	0	0	0	0	1	89	90	0.99
Total	145	79	123	78	50	96	572	
Producer`s Accuracy	0.90	0.81	0.82	0.45	0.80	0.93		
Overall Accuracy (%)								77

Table S7 Confusion matrix for wetlands of Mediterranean/Humid subtropical climate zone after applying RF

**Wetland class**	Marsh	Mangrove	Swamp	Peatland	Seasonal wetlands	Deep water	Total	User`s Accuracy
Marsh	31	1	2	2	3	0	39	0.79
Mangrove	1	17	5	2	0	0	25	0.68
Swamp	1	2	28	10	2	0	43	0.65
Peatland	2	3	11	29	0	0	45	0.64
Seasonal wetlands	10	0	2	4	43	4	63	0.68
Deep water	0	0	0	0	2	51	53	0.96
Total	45	23	48	47	50	55	268	
Producer`s Accuracy	0.84	0.86	0.95	0.80	0.89	0.98		0.88
Overall Accuracy(%)								73
